# Charcot Marie Tooth 2B Peripheral Sensory Neuropathy: How Rab7 Mutations Impact NGF Signaling?

**DOI:** 10.3390/ijms18020324

**Published:** 2017-02-04

**Authors:** Harry Liu, Chengbiao Wu

**Affiliations:** Department of Neurosciences, University of California San Diego, Medical Teaching Facility, Room 312 MC-0624, 9500 Gilman Drive, La Jolla, CA 92093-0624, USA; hul023@ucsd.edu

**Keywords:** CMT2B, peripheral sensory neuropathy, NGF, Rab7, mutations, axons, lysosomes, autophagy

## Abstract

Charcot-Marie-Tooth 2B peripheral sensory neuropathy (CMT2B) is a debilitating autosomal dominant hereditary sensory neuropathy. Patients with this disease lose pain sensation and frequently need amputation. Axonal dysfunction and degeneration of peripheral sensory neurons is a major clinical manifestation of CMT2B. However, the cellular and molecular pathogenic mechanisms remain undefined. CMT2B is caused by missense point mutations (L129F, K157N, N161T/I, V162M) in Rab7 GTPase. Strong evidence suggests that the Rab7 mutation(s) enhances the cellular levels of activated Rab7 proteins, thus resulting in increased lysosomal activity and autophagy. As a consequence, trafficking and signaling of neurotrophic factors such as nerve growth factor (NGF) in the long axons of peripheral sensory neurons are particularly vulnerable to premature degradation. A “gain of toxicity” model has, thus, been proposed based on these observations. However, studies of fly photo-sensory neurons indicate that the Rab7 mutation(s) causes a “loss of function”, resulting in haploinsufficiency. In the review, we summarize experimental evidence for both hypotheses. We argue that better models (rodent animals and human neurons) of CMT2B are needed to precisely define the disease mechanisms.

## 1. Introduction

Charcot Marie Tooth (CMT) neuropathies are clinically and genetically heterogeneous hereditary diseases with a prevalence of ~1/2500 [1,2,3,4,5,6,7,8,9]. CMT has many subtypes (CMT1-4, CMTX) that affect motor and/or sensory nerves resulting in progressive distal muscle weakness and atrophy, foot deformities, distal sensory loss [10], and decreased or absent tendon reflexes [11,12,13,14,15,16,17,18]. Approximately 40 genes/loci have been identified to be associated with CMT [1], and no effective treatments are presently available [7,19,20]. CMT1 is the demyelinating disease [1,7,20,21,22]. CMT2 displays prominent axonal dysfunction [23]. CMT3 leads to severe infantile demyelinating neuropathy [2,4,5,12,13,21,22]. CMT4 represents subtypes of autosomal recessive demyelinating motor and sensory neuropathies. CMTX is caused by a point mutation in the *connexin-32* gene located on the X chromosome [4,13,21,22].

CMT2 also has many subtypes (A, B, D, E, H, I) [1,2,6,12]. These subtypes are clinically similar and classified based on molecular genetic findings. Specifically, we discuss CMT2B, a hereditary peripheral sensory neuropathy characterized by distal sensory loss, muscular weakness, and recurrent foot ulcers. Onset typically occurs between the first to third decade of life [6]. Affected limbs are prone to muscle atrophy and soft tissue infections, often leading to necessary amputation. Variable motor involvement coupled with prominent sensory loss and associated ulcerations have made the distinction of CMT2B between a hereditary motor and sensory neuropathy (HMSN) and hereditary sensory and autonomic neuropathy (HSAN) difficult [14,24,25].

While the prevalence of CMT is about 40 per 100,000 individuals [1,26], the prevalence of the various CMT2 subtypes remains unknown. This is largely because other causative genes have yet to be accounted for [27]. In addition, cultural standards and socioeconomic restraints in certain population areas may prevent clinical manifestations from being reported and treated, such as the Chinese-associated CMT2B N161I mutation [28]. Furthermore, patients with CMT2B may be difficult to distinguish from those with HSAN1 caused by mutations in the *SPTCL1* genes [29].

## 2. Rab7 Mutations Are Associated with CMT2B

The primary pathological feature of CMT2B is chronic axonal degeneration caused by mutations in Rab7, a ubiquitously expressed GTPase that serves as the master regulator of vesicular trafficking, maturation, and fusion in the late endocytic pathway [30]. Primarily localized in acidic pre-degradative and degradative organelles, such as late endosomes, lysosomes, and autophagosomes, Rab7 presents on the cytosolic surface of the vesicle membrane and interacts with various downstream effectors to carry out its regulatory functions [31,32]. Specifically, Rab7 orchestrates the transition of early endosomes into late endosomes, and the subsequent degradation of their associated cargos. This includes the lysosome-mediated degradation of epidermal growth factor (EGF) and its receptor EGFR, nerve growth factor (NGF), and tropomyosin receptor kinase A (TrkA). It has, therefore, been proposed that neurodegeneration in CMT2B is attributable to disrupted neurotrophin trafficking by mutant Rab7 [33].

**Figure 1:**
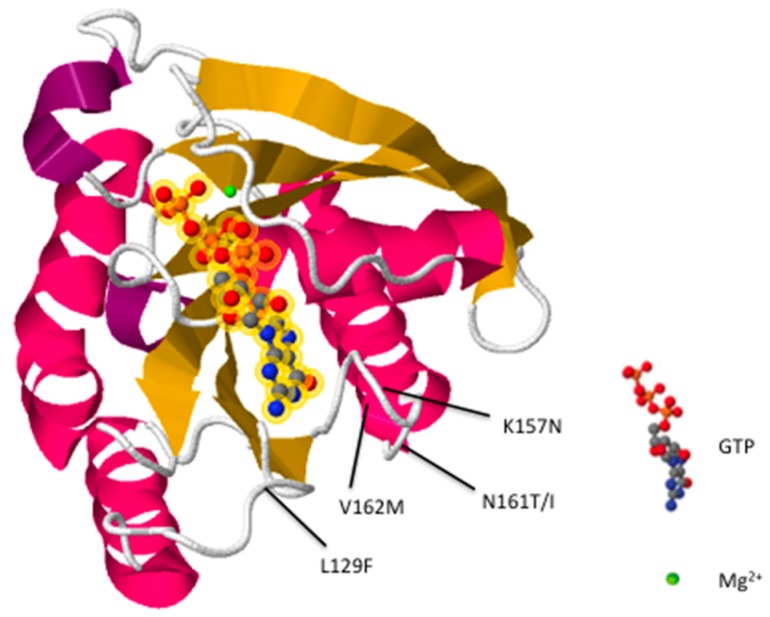
Predicted crystal structure of the Rab7 protein. α-helices are magenta/purple and β-sheets are yellow. Amino acid residues (L129, K157, N161, V162) affected by CMT2B mutations are indicated. The image was generated and modified using RCSB PDB structure 1T91 [42].

Conformational changes to the nucleotide binding pocket permit Rab7 to switch between its active (GTP bound) and inactive (GDP bound) forms. However, in CMT2B, five missense point mutations (L129F, K157N, N161T/I, and V162M) [17,28,34,35] occurring near the nucleotide binding pocket (Figure 1) decrease nucleotide affinity, causing unregulated nucleotide exchange [36,37,38]. The resulting mutants are more prone to bind GTP and behave similarly to constitutively active Rab7^Q67L^ [39]. Numerous studies have proposed that disease pathogenesis is attributable to increased Rab7 activity [33,37,38,39,40], suggesting treatment development should focus on inhibiting mutant pathways. However, some studies using *Drosophila* have proposed CMT2B pathology results from partial loss of Rab7 function [41]. To resolve this controversy, we conducted a comprehensive review of previous literature characterizing CMT2B associated Rab7 mutants. Ultimately, our findings suggest that CMT2B pathology is induced likely by overall gain of functionality in Rab7 mutants, characterized by excessive protein activation resulting in enhanced effector interactions and dysregulated endolysosomal transport.

## 3. Possible Pathogenic Mechanisms

All known CMT2B Rab7 mutations cause pathology in heterozygosity. As such, CMT2B is classified as an autosomal dominant disease [14]. Most dominant mutations lead to a gain of protein function, typically manifesting as increased activity, novel functionality, or abnormal expression of the gene product. However, some dominant mutations are associated with a loss of function; these mutations are typically dominant negative or result in haploinsufficiency. Prior experiments have demonstrated that CMT2B Rab7 mutations are not dominant negative. For example, CMT2B mutants can bind GTP similarly to wild-type and constitutively-active Rab7^Q67L^ [39]. On a cellular level, CMT2B mutants in HeLa cells coupled strongly with effector Rab-interacting lysosomal protein (RILP) to facilitate EGF degradation, while dominant negative Rab^T22N^ inhibited degradation due to weak interactions with RILP [39]. In addition, CMT2B and constitutively active Rab7 mutants reduced neurite outgrowth while dominant negative Rab7^T22N^ showed no significant effect both in vitro and in vivo in PC12 cells and zebrafish embryos [33,43]. In fact, inhibition of Rab7 activity by overexpressing Rab7^T22N^ was shown to trigger NGF-induced neurite outgrowth in PC12 cells [44]. While Rab7 CMT2B mutants are known to increase Erk1/2 phosphorylation in PC12 cells upon NGF-TrkA signaling, phosphorylated Erk1/2 was shown to accumulate in the cytosol rather than the nucleus, which could explain the inhibitory effect on neurite outgrowth [45]. Ultimately, these findings suggest CMT2B pathogenesis is likely caused by a gain of function in Rab7, and not attributable to dominant negative mutations.

Studies have also demonstrated that CMT2B pathology is not attributable to Rab7 haploinsufficiency. CMT2B mutants demonstrated active functionality by rescuing Rab7 function after endogenous Rab7 expression in transfected HeLa cells was silenced [39]. In addition, CMT2B mutant levels at and below endogenous Rab7 levels rescued Rab7 haploinsufficiency in *Drosophila* photoreceptor neurons [41]. Ultimately, the dominant nature and rescue of Rab7 function by CMT2B mutants support a gain of function characterization.

It is worth noting that although CMT2B Rab7 mutants show decreased general nucleotide affinity [37,39], there is little evidence to suggest an intrinsic GTPase defect or a net reduction in protein activation, which would support a loss of function hypothesis. Indeed, hydrolysis of radiolabeled GTP by CMT2B mutants occurred at significantly slower rates in competition with excess unlabeled GTP compared to wild-type Rab7 [37,39]. While this can be explained by an intrinsic GTPase defect, the results were ultimately attributed to an increased rate of GTP dissociation in CMT2B mutants, as demonstrated by partial rescue of the GTPase defect after omitting the competing unlabeled GTP from the assay [37]. Consistently, increased concentration of the radiolabeled GTP further restored GTPase activity in the same experiment, and a structural characterization of Rab7^L129F^ showed alterations to the nucleotide-binding pocket while conformations in catalytic sites were normal [37]. It is therefore worth emphasizing that reduced nucleotide affinity and a lower rate of hydrolysis per nucleotide binding event does not necessitate a net reduction in protein activation when GTP is in constant supply, as is the case in vivo [37]. In fact, dysregulated nucleotide exchange in CMT2B mutants resulted in an increased fraction of active, GTP-bound Rab7 [37,39], consistent with the gain of function characterization of CMT2B mutations.

As such, several previous studies have proposed gain of function mechanisms [33,37,38,39,40] to explain the dominant phenotypes of mutant CMT2B genes. Compared to wild-type Rab7, CMT2B mutants L129F and V162M showed increased interactions with its specific effectors, including dynein-dynactin recruiting RILP, vacuolar protein sorting-associated protein 13 (Vps13C), and cholesterol sensor oxysterol-binding protein-related protein 1L (ORP1L) [37,39] (Table 1). CMT2B mutants also showed stronger affinity for clathrin heavy chain, intermediate filament protein peripherin, and increased phosphorylation of vimentin in HeLa and Neuro2A cells compared to wild-type Rab7 [37,40,46]. There is also evidence that CMT2B mutants could interact more frequently with effector Rabring7, an ubiquitin ligase that regulates EGFR degradation. Upregulating Rabring7 activity was shown to increase perinuclear aggregation of lysosomes [47]. Expression of constitutively active Rab7 also led to clustering of late endosomes in the perinuclear region, which delayed entry of EGFR into late endosomes. This subsequently delayed EGFR degradation and led to a prolonged mitogen-activated protein kinase (MAPK) activation [48], consistent with the effect of CMT2B mutants, which activate Rabring7 in a nucleotide-dependent manner [45,47].

A potential consequence of increased Rab7-effector interactions could be altered axonal transport speeds of mutant-containing vesicles. Indeed, all CMT2B mutant-containing vesicles moved at faster anterograde speeds in rat DRG neurons compared to their wild-type counterpart [33]. In *Drosophila* sensory neurons and mammalian neuroblastoma neurites, axonal transport of mutant L129F-containing vesicles paused less often compared to wild-type vesicles [61]. Similarly, mutant N161T and V162M-containing vesicles paused less often in vertebrate zebrafish embryos [43]. However, the same study showed reduced vesicle transport speeds in L129F and K157N containing vesicles [43]. The contradictory findings from different models and cell types illustrate the diverse and complex nature of CMT2B Rab7 mutants and their effects on neurobiology. However, increased interaction with downstream effectors and altered vesicle transport are both attributable to Rab7 hyperactivity, collectively validating a gain of function characterization of CMT2B Rab7.

**Figure 2:**
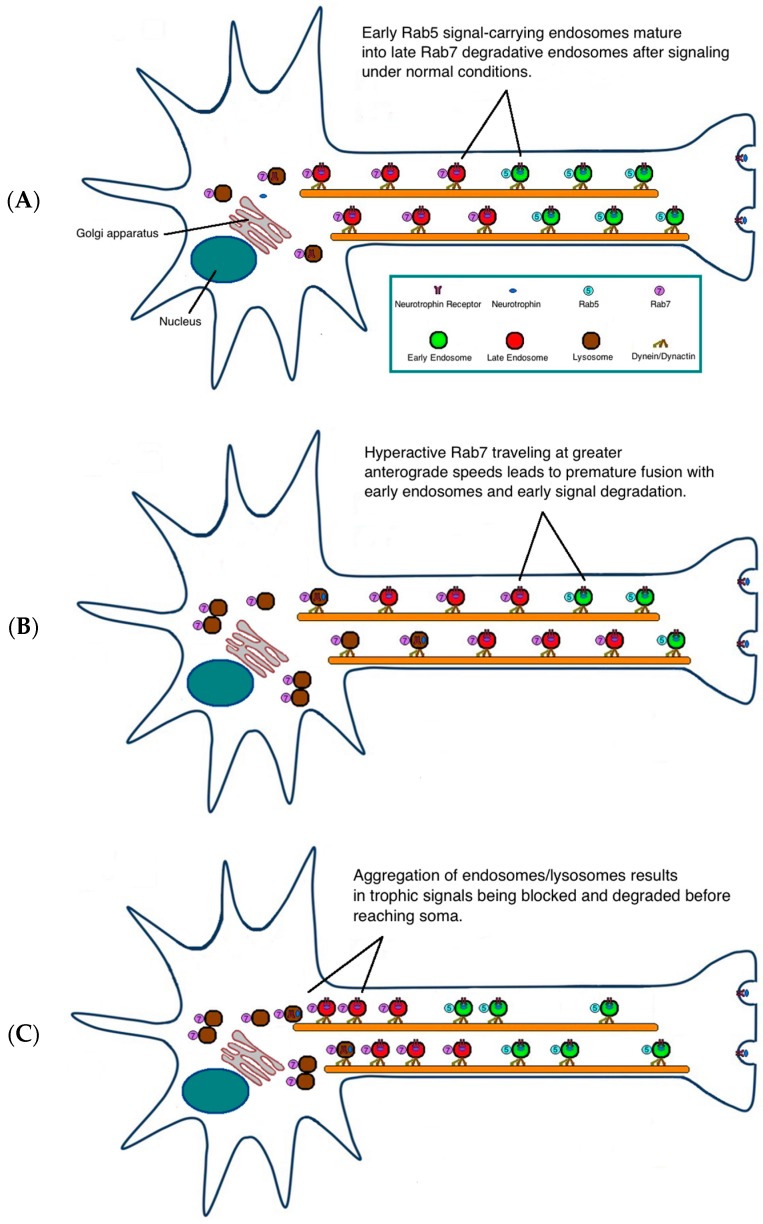
Proposed models of pathogenic mechanism. Under normal conditions, Rab5 and Rab7 deliver trophic signals to the soma under tight regulation. Upon delivery of the signal, Rab5-positive early endosomes transition to late endosomes/lysosomes facilitated by Rab7 (**A**). In CMT2B, hyperactive Rab7 vesicles with greater affinity for downstream effectors could travel at faster anterograde speeds, resulting in premature fusion and degradation of signal-carrying Rab5 endosomes (**B**). Alternatively, hyperactivation of Rab7 vesicles could lead to aggregation of late endosomes/lysosomes near the nucleus and in the axon, consequently blocking trophic signals from reaching the soma (**C**).

Variations among observed mutant phenotypes could exist due to differences in cell types, motor proteins, and sub-cellular signaling processes. Steric effects could also vary between different CMT2B mutations and differentially impact endosome dynamics. Indeed, Rab^K157N^ was the first CMT2B mutant associated with a loss of function. Studies have shown that K157N, unlike other CMT2B mutants, does not interact with the retromer complex [62,63]. This supports the postulate that a slower membrane cycling of CMT2B Rab7 mutants inhibits trafficking and degradation of endocytosed growth factor receptors [64]. The alternative hypothesis suggests that pathogenesis stems from more rapid degradation of the endocytosed growth factor receptors [33,38]. However, we propose that both postulations could be explained by a consensus increase in anterograde axonal transport speeds of CMT2B mutant-containing vesicles [33]. Unlike in normal neurons (Figure 2A), increased Rab7 activity and subsequent upregulated anterograde axonal transport of both degradative and non-degradative systems could prematurely degrade (Figure 2B) or hinder (Figure 2C) critical trophic signals from reaching the nucleus. Collectively, both hypotheses suggest that differential interactions of CMT2B mutants with their specific effectors disrupt the efficiency of normal endosomal protein sorting and trafficking. This would be particularly detrimental in the long axons affected in CMT2B patients [62].

An accurate functional characterization of CMT2B mutants is crucial to directing therapeutic development towards either inhibiting or upregulating endogenous Rab7 function in patients. More studies could be done to further test for gain of Rab7 functionality of CMT2B mutants. For instance, changes in effector interactions could be assessed for the homotypic fusion and protein sorting (HOPS) tethering/GEF complex, Vps35-Vps26-Vps29 core retromer complex, and Rabring7. In addition, overexpression of Rab7 was shown to reduce toxic cholesterol accumulation in Niemann-Pick type C cells [65]. As such, Rab7 overexpression could be replaced with endogenous levels of CMT2B mutants to see if a similar effect occurs. Gene targeting could also be used in future studies to compare phenotypes between heterozygous and homozygous CMT2B knock-ins, and to determine if *CMT2B* genes are haplosufficient. However, findings from previous studies encompass extensive genetic and biochemical analyses that strongly support a gain of function classification of CMT2B mutations. These results reaffirm the current consensus that future treatments should aim to ultimately inhibit Rab7 hyperactivity in affected patients.

## 4. Conclusions

CMT2B peripheral sensory neuropathy is a rare genetic disorder caused by single point mutations in Rab7. Since Rab7 is expressed ubiquitously, it is extremely intriguing why only peripheral sensory neurons, are affected in the disease. Current studies have indicated that enhanced lysosomal and autophagic activities are likely responsible for diminishing NGF trafficking and signaling and inducing axonal degeneration in CMT2B. A thorough understanding of the disease mechanisms will reveal the fundamental biology of Rab7. In addition, these efforts will have important implications in research of other neurodegenerative diseases, since Rab7 has also been implicated in Parkinson’s diease [66,67,68,69] and Niemann Pick disease [65].

## Figures and Tables

**Table 1:** Impact on effectors by Rab7 mutations in CMT2B.

Effector	Function	CMT2B Rab7	Reference
HOPS Complex	Tethering (regulates endosomal membrane fusion)/GEF	Possible decreased interaction. CMT2B mutants can exchange GTP in GEF-independent manner.	[30,37,49,50,51,52,53]
RILP/ORP1L	Recruit and activate dynein-dynactin motor complex. Regulate late endosome/lysosome organization and transport	Increased interaction shown in L129F and V162M mutants.	[37,54,55,56,57]
Vps13C	Vacuolar protein sorting-associated protein	Increased interaction shown in L129F and V162M mutants.	[37]
Retromer Core Complex	Regulates retrograde transport from endosomes to *trans*-Golgi network.	Possible increased interaction. Rab7 binds in nucleotide dependent manner.	[58,59]
Rabring7	Ubiquitin ligase that regulates EGFR degradation	Possible increased interaction. Rab7 binds in nucleotide dependent manner.	[45,47,48,60]
